# Fiber Optic Load Cells with Enhanced Sensitivity by Optical Vernier Effect

**DOI:** 10.3390/s21227737

**Published:** 2021-11-20

**Authors:** Tiago Paixão, Ricardo Ferreira, M. Fátima Domingues, Paulo Antunes

**Affiliations:** 1 I3N and Physics Department, Campus of Santiago, University of Aveiro, 3810-193 Aveiro, Portugal; ricardo.a.moreira@ua.pt (R.F.); pantunes@ua.pt (P.A.); 2 IT—Instituto de Telecomunicações, University of Aveiro, 3810-193 Aveiro, Portugal; fatima.domingues@ua.pt

**Keywords:** optical fiber sensors, load sensing, optical Vernier effect, Fabry–Pérot interferometer

## Abstract

Developing technologies capable of constantly assessing and optimizing day-to-day activities has been a research priority for several years. A key factor in such technologies is the use of highly sensitive sensors to monitor in real-time numerous parameters, such as temperature and load. Due to their unique features, optical fiber sensors became one of the most interesting and viable solutions for applications dependent on those parameters. In this work, we present an optical fiber load sensor, called load cell, based on Fabry–Pérot hollow cavities embedded in a polymeric material. By using the load cells in a parallel configuration with a non-embedded hollow cavity, the optical Vernier effect was generated, allowing maximum sensitivity values of 0.433 nm N^−1^ and 0.66 nm °C^−1^ to be attained for vertical load and temperature, respectively. The proposed sensor’s performance, allied with the proposed configuration, makes it a viable and suitable device for a wide range of applications, namely those requiring high thermal and load sensitivities.

## 1. Introduction

Optical fiber sensors (OFSs) have been used extensively in recent years in several fields (from biology to civil engineering), mainly due to their intrinsic advantages, such as their light weight, immunity to electromagnetic fields, multiplexing capabilities, electrical passiveness at the point-of-care, and multiparameter sensing [1,2]. Despite the numerous features of conventional OFSs, the limits of sensing resolutions are being reached. Therefore, efforts are being made to improve OFS resolutions, either by using new materials or adopting novel sensing configurations. Recently, one of the most attractive and commonly used techniques to increase the sensitivity of optical fiber sensors is the optical Vernier effect (OVE). Despite being solely used within interferometry-based OFSs, the inherent advantages of this effect, such as the possibility to tune OFSs’ sensitivities and develop highly compact devices, have allowed researchers to reach unprecedented sensitivity values, mainly in applications for temperature [3,4], strain [5,6], magnetic fields [6,7] and refractive index monitoring [8,9].

In this work, an optical fiber sensor, based on a hollow Fabry–Pérot interferometer (FPI), embedded in resin, for temperature and vertical load monitoring, is reported. The sensors’ sensitivity was magnified using the optical Vernier effect, resulting from coupling, in a parallel configuration, the embedded sensor with an additional FPI cavity insensitive to temperature variations, eliminating the need to adopt complex isolation schemes or mathematical compensation methods. The thermal and load characterization revealed a linear response throughout the entire tested ranges and a dynamic range up to a maximum value of approximately 150 N. The attained results, allied with the low-cost fabrication methods and devices’ compactness, unveil the enormous potential of this sensor’s configuration to be used in a wide range of different applications, acting either as a load cell or temperature sensor.

## 2. Materials and Methods

**Figure 1:**
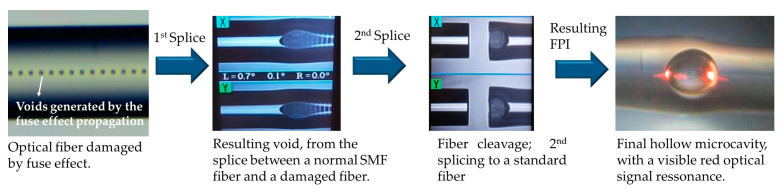
Hollow microcavity production by the recycling of optical fiber damaged by the catastrophic fiber fuse effect.

The developed sensor comprises two different FPIs, namely reference and sensing FPI (rFPI and sFPI, respectively). Both FPIs were fabricated based on the methodology previously reported by our team [10], which consists of the recycling of optical fibers previously damaged by the fiber fuse effect. This catastrophic phenomenon completely destroys the optical fiber along its path, leaving periodic voids along the optical fiber core, as visible in Figure 1, on the left. These periodic voids inhibit the optical fiber’s transmission abilities, rendering it useless for communications purposes. Nevertheless, its use for the production of inline Fabry–Pérot optical fiber interferometric sensors has been exhaustively explored [10,11,12]. The methodology to produce the FPI sensors is based on the machine splicing technique and is also depicted in Figure 1: It starts with the normal splice between the damaged fiber and the normal SMF fiber, which results in a void with higher dimensions in the splice region. The following step is to cleave this fiber in the splice region in order to isolate the void with higher dimensions. Afterwards, this fiber is spliced again to another SMF fiber, which results in a hollow microcavity, as depicted in Figure 1, on the right. The size of the resulting microcavity can be controlled by the size of the void in the 2nd splice (which is set by the cleave point).

**Figure 2:**
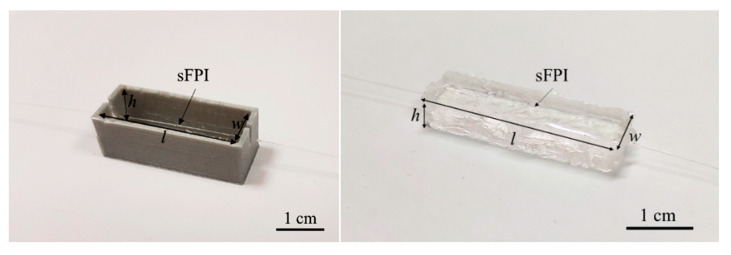
Load cell production apparatus and the resulting load cells. sFPI stands for the sensing FPI, which comprises the two different load cells (LC_1_ or LC_2_), and *l*, *h* and *w* are the cell’s length, height and width, respectively.

Assembling the load cells comprised of the encapsulation of the FPIs (one for each pair of rFPI and sFPI). Two 3D printed molds with a different height (*h*) and width (*w*) were used (the length (*l*) was kept constant) to evaluate the influence of the resin volume on the sensitivity of the load cells. The optical fiber containing the hollow microcavity was placed at the center of the cell’s cast, which was then filled with a thermal setting epoxy resin (Liquid Lens), as depicted in Figure 2. The resin was left to cure for 24 h to guarantee its full solidification, after which the resin block with the embedded optical fiber (load cell) was extracted from the mold. The used molds, as well as the resulting structures, are depicted in Figure 2.

To generate the OVE, one needs to overlap two interferometric signals, either in series or parallel configuration, with slightly detuned frequencies. Such superposition gives origin to a beating shape spectrum, which can usually be modulated by an envelope function [13,14]. Typically, one can fully describe the OVE envelope in terms of its free spectral range (FSR_env_). Assuming the specific case of OVE generation by two FPIs in a parallel configuration, where one acts as reference (rFPI) and the other one as sensing element (sFPI), the FSR_env_ is given as [4,6]:

(1) $${\mathrm{FSR}}_{\mathrm{env}}=\left|\frac{{\mathrm{FSR}}_{r}{\mathrm{FSR}}_{s}}{{\mathrm{FSR}}_{r}-{\mathrm{FSR}}_{s}}\right|,$$

where FSR_r_ and FSR_s_ are the free spectral ranges of rFPI and sFPI, respectively. The FSR of each FPI can be written as [13,15]:

(2) $${\mathrm{FSR}}_{r,s}=\frac{\lambda_{1}\lambda_{2}}{2{\mathrm{nL}}_{r,s}},$$

λ_1,2_ being the central wavelengths of two adjacent maxima/minima, and n and L the FPIs’ cavity medium refractive index and physical length, respectively. Considering the rough approximation $\lambda_{1}\lambda_{2}\approx \lambda^{2}$, Equation (2) can be further simplified:

(3) $${\mathrm{FSR}}_{r,s}=\frac{\lambda^{2}}{2{\mathrm{nL}}_{r,s}},$$

**Figure 3:**
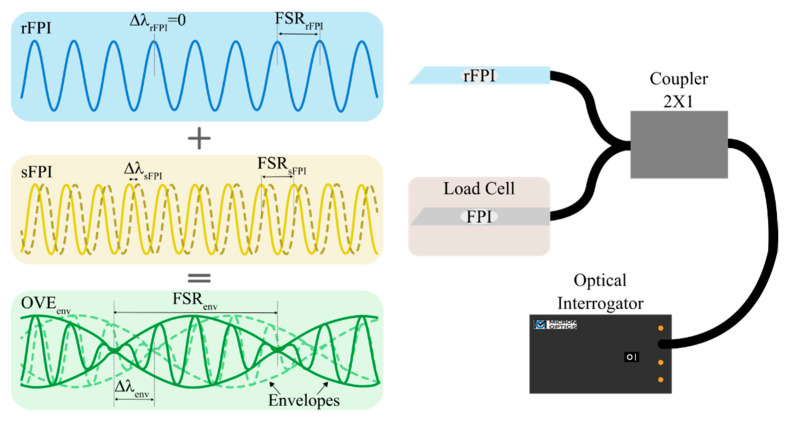
Schematic representation of the OVE-based load cells’ spectral monitoring mechanism in a parallel configuration. The solid and dashed lines represent the spectra before and after applying an external stimulus, respectively.

Assuming that rFPI and sFPI reflection spectra (R_r,s_) can be mathematically described as [4,6]:

(4) $$R_{r,s}=a_{r,s}\cos\left(\frac{4{\pi L}_{r,s}}{\lambda }\right),$$

where a_r,s_ is an arbitrary reflection amplitude, the OVE spectrum shall be given by the sum of the two FPIs’ spectra, as schematically represented in Figure 3.

One of the most important figures of merit of the OVE is the magnification factor (M). By definition, M represents the sensitivity enhancement ratio between sFPI and the OVE envelope. Considering S_sFPI_ and S_env_ as the sensitivities of sFPI and the OVE envelope, respectively, M can be written as:

(5) $$M=\frac{S_{\mathrm{env}}}{S_{\mathrm{sFPI}}}=\frac{\Delta \lambda_{\mathrm{env}}}{\Delta \lambda_{\mathrm{sFPI}}},$$

where Δλ_sFPI_ and Δλ_env_ are the wavelength shifts of each spectrum when an external stimulus is applied.

As depicted in Figure 3, if rFPI is insensitive to the desired measurand, or if it is maintained at constant and controlled conditions, a small wavelength variation experienced by the sFPI originates an enormous wavelength shift of the OVE_env_. This principle is called the traditional OVE [16] and was applied to the sensors developed in this work, using the two fiber optic hollow cavities embedded in an epoxy resin (denominated as LC_1_ and LC_2_) as sFPIs, and as rFPI, a similar fiber optic hollow cavity, but without any embedding material.

**Figure 4:**
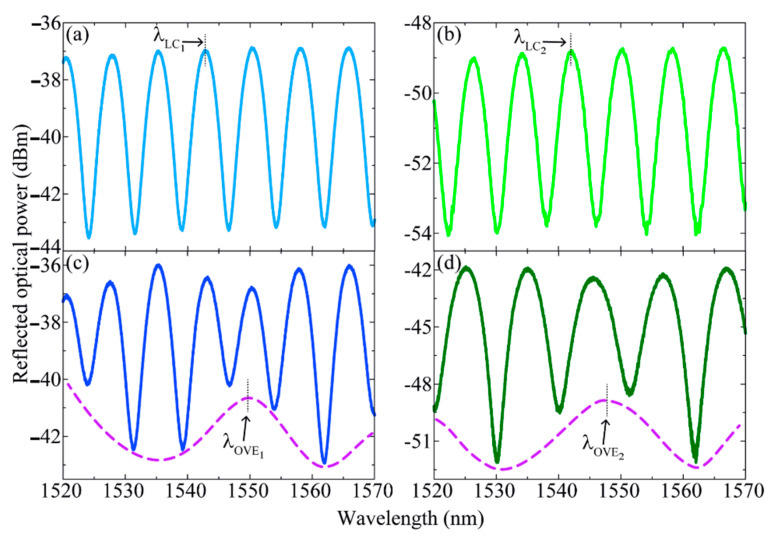
Spectra of (**a**) LC_1_, (**b**) FPI_2_, (**c**) VFPI_1_ and (**d**) VFPI_2_. The purple dashed lines in (**c**,**d**) represent the lower envelopes of the spectra. λ_LC1,2_ and λ_OVE1,2_ are the monitored central wavelengths of the FPIs and OVEs’ envelopes, respectively, used in the sensors’ characterizations.

Two different load cells (LC_1_ and LC_2_) were used to assess how the volume distribution of the embedding resin affects the overall load cells’ sensitivity. The final dimensions of LC_1_ and LC_2_ are presented in Table 1, and the spectra of LC_1_ and LC_2_ (measured in reflection by a Micron Optics optical interrogator, model SM125 with 5 pm of resolution and an acquisition rate of 1 Hz), as well as the respective OVE spectra generated in a parallel configuration with the rFPI (denominated OVE_1_ and OVE_2_, respectively), are depicted in Figure 4. 

**Figure 5:**
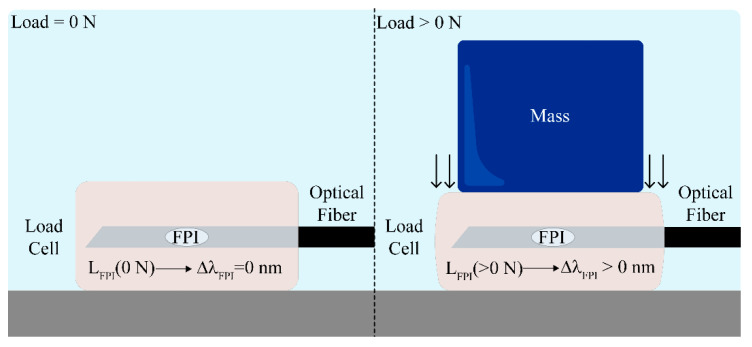
Schematic representation of the load cells under different load forces. For different applied mass, the FPI cavity longitudinal length (L_FPI_) suffers a variation, which translates into a spectral wavelength shift (Δλ_FPI_).

When a vertical load is applied to each cell, the embedding resin will contract along the Z axis and expand in the remaining directions (*X* and *Y* axis). Therefore, a spectra wavelength shift is expected for LC_1_ and LC_2_ and, consequently, in OVE_1_ and OVE_2_ as well. Figure 5 depicts a schematic of the forces involved when a mass is placed on the top of one of the developed load cells, where L_FPI_ represents the embedded FPI cavity length, and Δλ_FPI_ the correspondent wavelength shift.

## 3. Results

To characterize the developed sensors in terms of temperature and vertical load, two separated setups were assembled. 

**Figure 6:**
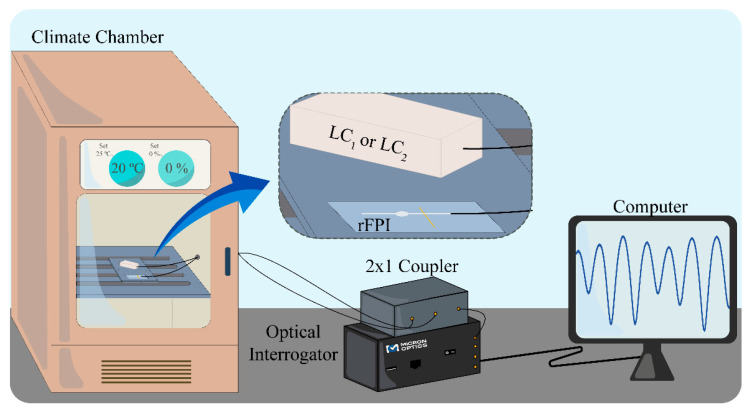
Schematic representation of the setup used in the thermal characterization. The zoom-in depicts the positioning of LC_1_ (or LC_2_) and rFPI inside the climate chamber.

**Figure 7:**
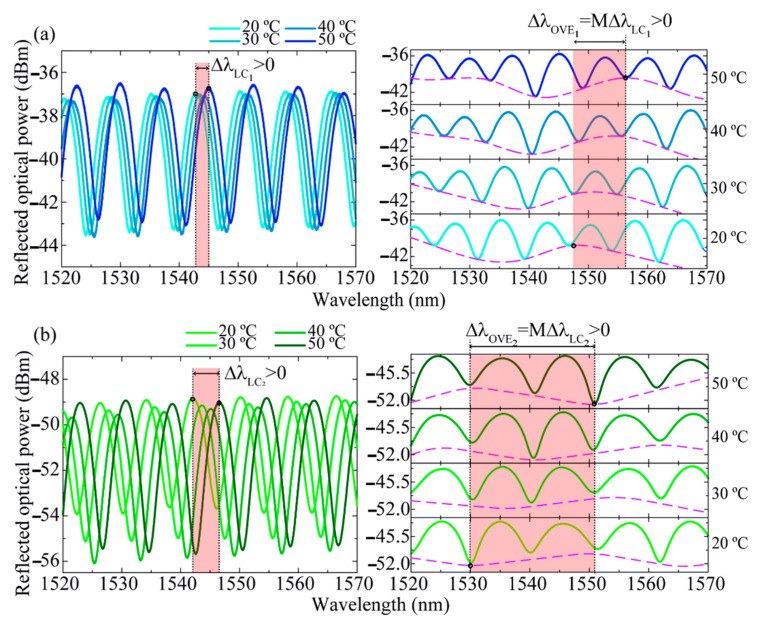
Spectra of (**a**) LC_1_/OVE_1_ and (**b**) LC_2_/OVE_2_ for four different temperature values. On the right, the purple dashed lines represent the lower envelopes of the OVE-based sensors.

First, the two sensors were thermally characterized using a climatic chamber (Weiss Technik L C/64/70/3, 0.3 °C of resolution). The temperature was varied in steps of 5 °C within the range of 15–50 °C, with a stabilization time of 15 min at each step. As already reported in the literature, fiber optic FPIs with an air cavity similar to the one of the rFPIs can be considered insensitive to temperature for small thermal variations [17,18]. Therefore, the sensing configuration depicted in Figure 6. was adopted, where both LCs were placed inside the climatic chamber along with the rFPI, which was fixed in a glass microscope slide with Kapton tape and left free of applied strain. At each temperature step, the individual spectra of LC_1_ and LC_2_ were recorded, as well as OVE_1_ and OVE_2_ spectra. A red shift was verified for all sensing elements, as can be seen in Figure 7. 

**Figure 8:**
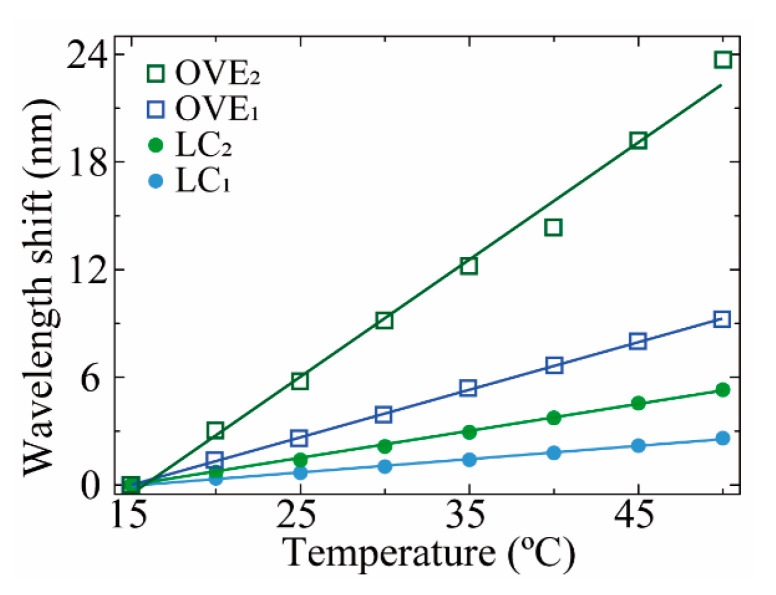
The wavelength shift of LC_1_ (closed blue circle), LC_2_ (closed green circle), OVE_1_ (opened blue square), and OVE_2_ (opened green square) with temperature. The colored solid lines represent the linear fits applied to each respective dataset.

The peaks’ central wavelength shifts of LC_1_/LC_2_ and OVE_1_/OVE_2_ with temperature variations were monitored, resulting in the data presented in Figure 8.

By applying linear fitting to the data presented in Figure 8, the maximum thermal sensitivity (S_T_) of 0.66 ± 0.03 nm°C^−1^ was attained with OVE_2_, representing a magnification factor value of M = 4.3 ± 0.3 relative to the sensitivity value achieved with the LC_2_ (0.153 ± 0.002 nm°C^−1^, Table 2). It should be noted that the sensitivities observed for LC_1_ and LC_2_ are due to their resin encapsulation: with the thermal variations, the resin will expand/contract, inducing a correspondent strain in the hollow cavity and correspondent wavelength shift of the FPI spectra.

**Figure 9:**
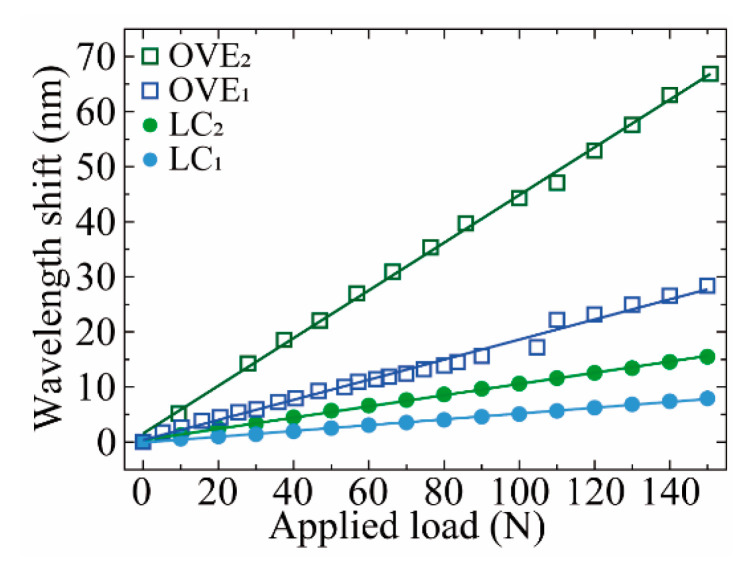
Wavelength shift of LC_1_ (closed blue circle), LC_2_ (closed green circle), OVE_1_ (opened blue square) and OVE_2_ (opened green square) with applied load force. The solid lines represent the linear fits applied to the respective data.

To assess the sensors’ responses to vertical load, the developed sensors were mounted on a mechanical test machine (Shimadzu^®^, AGS-5 kND). The spectral responses of both sensing elements (LC_1_ and LC_2_) were monitored for a load range of 0–150 N, approximately, resulting in the data plotted in Figure 9.

Analogous to the temperature characterization, the linear fits applied to the vertical load characterization data revealed that OVE_2_ attained the highest load sensitivity value (S_Load_ = 0.433 ± 0.005 nm N^−1^), which translates to a magnification factor of M = 4.2 ± 0.1 (Table 2), and presented a high linear correlation factor (R^2^ > 0.998). The higher sensitivity values of OVE_2_ may be explained not only by the magnification provided by the optical Vernier effect but may also be due to the sensing FPI intrinsic physical characteristics, namely its dimensions and shape. As temperature and load variations are transduced as strain to the FPI cavities, it is known that the larger the FPI physical length is, or the lower FSR value (in this case, FSR_LC1_ ≈ 7.67 nm and FSR_LC2_ ≈ 8.13 nm), the less sensitive to strain variations it will be, as suggested in [19].

From the results presented in Table 2, it can be verified that all load cells’ configurations present linear behavior for both temperature and normal load variations. However, slight structural differences in the sensing elements (volume of load cells and physical dimensions of the sFPIs) may lead to major sensitivity discrepancies, even if the same resin material is used, as occurred in LC_1_ and LC_2_. Therefore, since similar magnification values were provided to OVE_1_ and OVE_2_ by the optical Vernier effect, the higher sensitivities achieved by OVE_2_ were expected. The maximum theoretical resolutions were calculated for this device, attaining the values of 0.008 °C and 0.012 N for temperature and load, respectively, determined by dividing the optical interrogator wavelength resolution by the attained sensitivities. The results presented in this work corroborate the use of the proposed sensor scheme to monitor temperature and normal load variations, with a sensitivity performance comparable with works already reported in the literature [17,18,20], but for a wider range of applied normal load (0–150 N).

## 4. Conclusions

In summary, a novel optical fiber sensor architecture for vertical load sensing was developed based on hollow FPI cavities embedded in epoxy resin (LiquidLens Advanced). The highest load sensitivity values were attained for the load cell with higher encapsulating dimensions (LC_2_), corroborating the use of similar physical parameters to develop new load cells in the future. By coupling the LC_1_ and LC_2_ cavities with a similar non-embedded one in a parallel configuration, the optical Vernier effect was generated, attaining a maximum vertical load sensitivity of 0.433 nm N^−1^, which is ~4.2 times higher than the sensitivity value of the single LC_2_ FPI sensor (0.102 nm N^−1^). Despite presenting relatively high-temperature sensitivities, in the future, cross-sensitivity issues could be mitigated by using both load sensors simultaneously and the respective 2 × 2 sensitivity matrix or by using another temperature sensor as a reference. As the rFPI used to generate the OVE is insensitive to temperature, the sensing architecture complexity was further reduced since mathematical compensations and isolation schemes are not required. Therefore, the small footprint and high sensing resolutions of the proposed sensors make them a suitable and valid solution for many sensing challenges, especially for those where very sensitive and compact load sensors are required.

## Figures and Tables

**Table 1:** Physical dimensions of the load cells LC_1_ and LC_2_, where w, h and l are the width, height and length of each cell, respectively.

Sensor	w (cm)	h (cm)	l (cm)
LC_1_	1.00 ± 0.05	0.40 ± 0.05	3.00 ± 0.05
LC_2_	1.70 ± 0.05	0.90 ± 0.05	3.00 ± 0.05

**Table 2:** Summary of the LC_1_, LC_2_, OVE_1_ and OVE_2_ sensitivities to temperature (S_T_) and normal load (S_Load_) variations.

Sensor	S_T_ (nm °C^−1^)	R^2^	M-Factor	S_Load_ (nm N^−1^)	R^2^	M-Factor
LC_1_	0.074 ± 0.001	0.998	--	0.053 ± 0.001	0.999	--
LC_2_	0.153 ± 0.002	0.999	--	0.102 ± 0.001	0.999	--
OVE_1_	0.265 ± 0.002	0.999	3.6 ± 0.1	0.182 ± 0.004	0.988	3.4 ± 0.1
OVE_2_	0.66 ± 0.03	0.988	4.3 ± 0.3	0.433 ± 0.005	0.998	4.2 ± 0.1

## Data Availability

Not applicable.
